# Rice bran mineral extract increases the expression of anagen-related molecules in human dermal papilla through wnt/catenin pathway

**DOI:** 10.1080/16546628.2017.1412792

**Published:** 2017-12-05

**Authors:** Yu-Mi Kim, Soon-Joung Kwon, Hyun-joon Jang, Young-Kwon Seo

**Affiliations:** ^a^ Department of Medical Biotechnology (BK21 Plus team), Dongguk University, Seoul, Korea

**Keywords:** Rice bran mineral extracts, dermal papilla, hair loss prevent, wnt-3α and β-catenin

## Abstract

The objective of this study is to evaluate of rice bran mineral extract (RBM) increases the expression of anagen-related molecules in human dermal papilla (DOCs). Four treatment groups were established to evaluate the efficacy of RBM, including a negative control, positive control (ascorbic acid), RBM and ortho-silicic acid (Si(OH)_4_) (OSA) group. Three days after the DPCs were administered the various treatments, western blot analysis showed that type I collagen expression was increased 2.5-fold in the OSA group and 4-fold in the RBM group, and ALP expression was increased 1.5-fold in the OSA and RBM group while the expression of fibronectin was increased ~3-fold in the OSA group and 2.5-fold in the RBM group. Also, the expression of Wnt-3α and β-catenin protein was increased in OSA and RBM group compared to control group. Furthermore, the expression of IL-1a was decreased by more than 50% in the OSA and RBM groups compared to the negative control. Analysis of mRNA expression by RT-qPCR showed that type I collagen increased 1.2-fold in the OSA- and RBM-treated DPCs, whereas type IV collagen increased 2.7-fold in the OSA group and 3.5-fold in the RBM group. However, TGF-β2 mRNA decreased about 80% in the OSA and RBM groups, respectively. Immunohistochemical staining of the DPCs for versican protein showed a significant increase in the OSA- and RBM-treated groups compared to the negative control. Thus, RBM have a potential to recover of DPCs activity and decreased inflammatory-related markers. It can be expected that hair loss prevention and hair growth enhancement can be expected when RBM is applied as a cosmetic product.

## Introduction

Human hair dermal papilla cells (HHDPCs) are essential for both the development and formation of the hair follicle and constitute a reservoir of cells with the potential to differentiate into diverse cell types, such as muscle cells, adipocytes, fibroblasts and Schwann cells [1]. Furthermore, many studies with animal models indicate the interest in dermal papilla cells (DPCs) as a crucial cell subpopulation of the hair follicle in the regenerative processes of the skin. HHDPCs have also been postulated as an appropriate source for the construction of bioengineered skin substitutes, in cases where autografting is not a suitable option [1].

Considerable research has focused on the therapeutic potential of natural products to stimulate hair growth. For instance, *Allium cepa* and *Ziziphus jujuba* extracts promoted hair growth and this was attributed to the antioxidant capacities of the respective extracts[2]. Hideaki et al showed that Lygodii Spora ethanol extract had significant anti-androgenic activity in testosterone-sensitive mice and on the growth of pigmented macules of the hamster ﬂank organ [3]. Adhirajan et al confirmed that the petroleum ether extracts of *Hibiscus rosa-sinensis* Linn. flowers and, particularly leaves, stimulated hair follicle growth *in vitro* and in a rat model [4]. Also, Lee et al verified *Rumex japonicus* Houtt. extract activated a hair growth marker in DPCs and promoted hair growth in mice [5]. Seeds of *Delphinium* species have being used for hair enhancement and treatment of hair loss since ancient times, particularly in Mediterranean traditional medicine. Koparal et al demonstrated that vinegar and water extracts of *Delphinium staphisagria* promoted angiogenesis *in vitro* in human keratinocyte cells and human umbilical vein endothelial cells, suggesting the potential to promote hair growth [6].

Rice bran extract (RBE) has the ability to differentiate cells [7] and suppress the proliferation of human colon cancer cells [8]. It can also increase mitochondrial function in neural cells [9] and displays an inhibitory effect on melanogenesis by downregulation of MITF [10]. Additionally, rice bran possesses many phytochemicals and nutrients, with known chemopreventive and immune-enhancing properties, such as vitamins, γ-oryzanol, tocopherols, tocotrienols, phenolic acids, carotenoids, polysaccharides, phytosterols, essential amino acids, and micronutrients. Choi et al. showed that the linoleic acid and γ–oryzanol components in supercritical carbon dioxide RBE promoted hair growth by inducing hair follicles into the anagen stage (active growth phase) and increasing the expression of associated cell growth factors in mice [11].

Rice bran ash, the end-product of rice bran incineration, is rich in silicon (Si, 65 wt%), but also contains other minerals, such as potassium (K), phosphorous (P), carbon (C), K_2_O, CaO, Na_2_O, MgO, Al_2_O_3_, ZnO, MnO_2_, and Fe_2_O_3_, as well as soluble silicic acids [11]. Silicon (Si) is the second most abundant element (27.2%) present in the earth crust and. it is known for a number of crucial chemical and physical properties, e.g. as a semiconductor [12]. Most silicone compounds are water-insoluble, causing cytotoxicity *in vivo*. However, the silicic acid anhydride of monomeric ortho-silicic acid (H_4_SiO_4_, OSA) is water-soluble and stable in highly diluted aqueous solutions [12]. Moreover, it has low toxicity compared to insoluble silicic acid. Oral intake of choline-stabilized OSA (ch-OSA), a bioavailable form of Si, was found to have a significant positive effect on brittleness of hair and nails and on hair morphology and tensile strengths [12]. Also, Grotheer et al. showed that OSA had anti-inflammatory properties and aided chronic wound recovery [11]. Nonetheless, research on hair growth using rice bran mineral (RBM) extract has not yet been well-studied. In this study, we researched the effect of RBM on DPCs. RBM and OSA were added to DPCs for 3 days and the DPC activity was evaluated by western blot, reverse transcription polymerase chain reaction (RT-qPCR), and immunohistochemical staining.

## Material and methods

### RBM extract and OSA

RBM was obtained from the carbonized chaff of rice bran (Japonica, Oryza sativa). Briefly, a 200-g aliquot of rice bran ash was added to 1 L distilled water and stirred at 400 rpm at 100°C for 24 h. And, the mixture was filtered through 10-μm filter paper, centrifuged at 22g for 30 min at 25°C to remove any remaining particulates to obtain the supernatant of 800 ml. Next the supernatant was concentrated to 10-folds and then sterilized by filtration through a 0.2-μm syringe filter to obtain the final RBM extract of 80 ml. OSA was obtained from Natural Factors (Biosil, USA) [11]. The chemical composition of RBM is shown in Table 1.

**Table 1.** Chemical composition of RBM.

Element	Si	Ca	Mg	K	Na	P
mg/kg (solution)	180	5	17	2930	113	300

**Figure 1.**
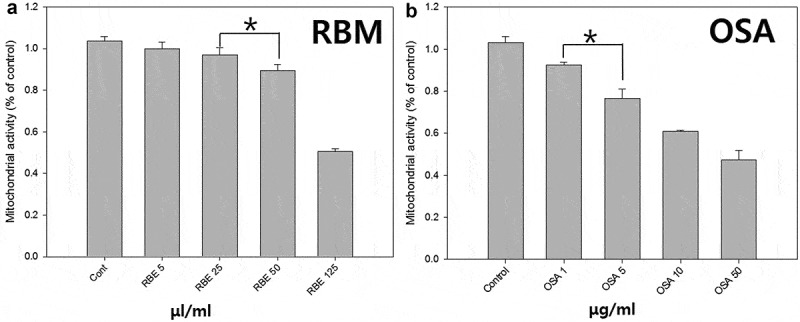
MTT analysis of rice bran extract and Biosil for concentration. The concentrate rating cell toxicity of rice bran extract (A) and ortho-silicic acid (B). (*p < 0.05).

The RBM extract and OSA were evaluated by the MTT (3-[4, 5-dimethylthiazol-2-yl]-2, 5 diphenyl tetrazolium bromide) assay for its effects on cell survival/proliferation (Figure 1).

### Dermal papilla (DP) cultures

The hair remaining after hair transplantation was collected following approval by the institutional review board (DUIRB-20151127–011). Hair follicles were obtained from the hair transplantation surgery with the patients’ approval. The tissue was washed in Dulbecco’s phosphate balanced solution (D-PBS) supplemented with penicillin G, streptomycin, and amphotericin B. Hair follicles were delivered to the laboratory in Williams E medium, containing 200 unit/ml penicillin G, 0.2mg/ml streptomycin, and 0.5 μg/ml. amphotericin B.

Using a stereoscope at 40x magnification (KSZ, Korea), the hair follicle bulb was sliced off and a 26 G syringe needle was used to isolate the DP and dermal sheath (DS) by softly teasing out the papilla. Then the basal stalk was cut off and the dermal sheath cells (DSCs) isolated. Finally, the papilla was transferred to a 35-mm culture dish that had been pre-coated with 400 µl FBS. And then Dulbecco’s minimal essential medium (DMEM, 2 ml) was added to reach a final 20% FBS. The media was fixed while the cells grew from the attached tissue. Once grown cells were observed, the FBS concentration was reduced to 10%. The media was changed twice a week. A 0.05% trypsin/0.02% EDTA solution was used to subculture confluent cells.

All cell media was replaced every 2 days. DPCs were subcultured when they reached 80 ~ 90% confluence in a 100Φ culture dish, with sub-culturing usually performed 1 to 3 times. The cells were washed with PBS to remove the FBS, dissociated using accutase (Innovative Cell Technologies, California, USA) and then incubated at 37°C, in a humid atmosphere of 5% CO_2_ for 5 ~ 7 min. Next, the accutase was collected and removed from the cells by centrifugation at 0.25g rpm for 5 min. The supernatant was discarded and the cell suspension inoculated into fresh cell culture media (DMEM containing 10% FBS, 100 unit/ml penicillin G, 0.1mg/ml streptomycin, and 0.25 μg/ml amphotericin B) and seeded in a 100Φ culture dish.

### Expose of the cells to UV-B (312 nm)

The DP cells were seeded at 0.5 × 10^5^/well in a 6-well plate (n = 3). After 24 h, the cell was exposed with UV-B. Prior to irradiation, cells were washed with PBS and irradiation was performed through a 1ml of PBS via exposure to 20 mJ/cm^2^ of UV-B at 312 nm dose, as measured with an SX-312 research radiometer (UVitec. Ltd.,). Irradiation time (15 sec) was estimated with radiometer until the dose of 20 mJ/cm^2^.

After irradiation, for the MTT and LDH assay, cells were cultured in DMEM without 10% FBS containing 30 μl/ml RBM or 2.4 μg/ml OSA or 50 μM of ascorbic acid 2-phosphate (As-2P, positive control) for 72 h. Only irradiation group was negative control.

Also, DP cells were cultured in DMEM with 10% FBS containing RBM or OSA or 50 μM of As-2P for 72 h for the apoptosis assay, RT-qPCR, western blot and immunohistochemical staining

### MTT assay for OSA and RBM

The MTT (Sigma) assay was performed to measure the usable concentration range of the RBM. Briefly, in 48-well plates, MTT solution (3 mg/ml) (n = 3) was injected into the cultured cells, followed by incubation at 37°C, in a humid atmosphere containing 5% CO_2_, for 90 min in the dark. The supernatant was then removed. Dimethyl sulfoxide was added to each well and the plates were shaken for 5 min before the absorbance was measured at 570 nm.

### Lactate dehydrogenase (LDH) assay

Cytotoxicity was evaluated using an LDH-LQ kit (Asan Pharmaceutical, Korea). Briefly, aliquots (100 μl) of the media obtained after culturing the cells for 3 days were placed in a 96-well plate, 50 μl of the working solution was added and then the plates were incubated at room temperature for 30 min. The reaction was terminated by adding 50 μL 1 N HCl and the absorbance was measured at 570 nm.

### Apoptosis assay

After three days with As-2p, OSA and RBM, cell apoptosis was measured using APOPercentage^TM^ assay kit (Biocolor Ltd. Antrim, UK) according to the manufacturer’s instructions. Colorimetric analysis (550-nm absorbance) was performed using a Victor plate reader (PerkinElmer Life Science, Turku, Finland).

### Western blotting

Cells were lysed in sample buffer (2% SDS, 0.1 mg/ml bromophenol blue, Tris-HCl pH 6.8, 5% 2-mercaptoethanol and 10% glycerol), and boiled at 100°C for 5 min. For the one of experimental group, each cells of 6-well plate were recovered with 200 μl sample buffer and accumulate. Protein was quantified using the bicinchoninic acid (BCA) (Thermo Scientific) assay after addition of sample buffer. The samples were loaded on 10% SDS-polyacrylamide gel electrophoresis (3.3 ml of 30% acrylamide/Bis Solution (BIO-RAD, CA, USA), 2.5 ml of 1.5 M Tris Glycine buffer (pH 8.8, Dyne Bio, Korea, Gyeonggi-do), 0.1 ml of 10% SDS solution (Dyne Bio, Korea, Gyeonggi-do), 0.1 ml of 10% ammonium persulfate solution (Dyne Bio, Korea, Gyeonggi-do), 4 ul of N,N,N’,N’-Tetramethylethylenediamine (Biosesang, Korea, Gyeonggi-do) and 4.0 ml of H_2_O), then the separated proteins were transferred to a nitrocellulose membrane (BIO-RAD, CA, USA), and then incubated with primary antibodies recognizing β-actin (Sigma–Aldrich, St Louis, USA), type I collagen, fibronectin, β-catenin, wnt3a and interleukin-1α (IL-1α) (Abcam, Massachusetts, USA). Protein detection was performed with Lumi Femto (Daeil Laboratory Service, Seoul, Korea) and the Molecular Imager ChemiDoc XRS+ station. Image J software (National Institutes of Health, Bethesda, MD, USA) was used to analyze and quantify the western blot image.

### Rt-qpcr

Total RNA was prepared from cultured cells using 500 μl TRIzol reagent (Invitrogen, USA). After solubilization, 100 μl chloroform (Sigma) was added, the mixture vortexed for several times and centrifuged at 25g, at 4°C for 15 min. The supernatant was transferred to a fresh tube and 500 μl isopropanol was added. After mixing and incubation at room temperature for 5 min, the solution was centrifuged at 35g at 4°C for 10 min. The supernatant was removed, and 1 mL of 70% ethanol added to the pellet, followed by centrifugation at 14g at 4°C for 5 min. The supernatant was discarded and the pellet dried at room temperature. Then, 20 μl diethylpyrocarbonate (DEPC)-water was added and the solution placed on ice. The nanodrop device was used to measure the purity and concentration of total RNA (Thermo Fisher Scientific, USA). An RT-PCR kit (Clontech, Palo Alto, CA, USA) was used to synthesize cDNA. The synthesized cDNAs were used in PCR reactions, in accordance with the manufacturer’s protocols using specific primers (Bioneer, Daejeon, Korea) for type I collagen, type IV collagen and TGF-β2 (Table 2). Band images were obtained with Molecular Imager ChemiDoc XRS+ (Bio-Rad, Hercules, CA, USA). For the quantitative analysis of the RT-PCR band images, Image J software (National Institutes of Health, Bethesda, MD, USA) was used

**Table 2.** Primers for the examined gene.

Gene	Primer 5ʹ	Primer 3ʹ	Size of product (bp)
TGF-β2	TCCGCACCCGAGACTGAC	AGGCTGAGCGCGACCGTG	441
Col 1a1	TCCCCAGCCACA AAGAGT	CGTCATCGCACA ACACCT	256
Col 4a1	GGCCCCTGCTGAAGCGTT	GATTCCACGAGCCCCTTG	342
GAPDH (control)	TGAAGGTCGGAGTCAACGGATTTGGT	CATGTGGGCCATGAGGTCCACCAC	983

### Immunohistochemistry

Immunohistochemistry was performed on cultured DP fixed at 4ºC for 1 h using 10% neutral buffered formalin. Endogenous peroxidase activity was blocked using 0.03% hydrogen peroxide, acted on the nonspecific reaction of bovine serum albumin and non-immune serum with the respective monoclonal antibody of versican (Thermo Fisher scientific, USA). Finally, it was acted on the standard immunohistochemical procedure with anti-goat or anti-rabbit immunoglobulin, using the avidin-biotin-peroxidase complex (ABC) method.

## Result and discussion

### Effect of RBM and OSA concentration on DP viability

To examine the effects of concentration of RBM and OSA on DP viability, the MTT assay was performed after cultures of 3 days. The initial seeding cell number in each culture was the same (5 × 10^4^ cells/well) in each group. As shown in Figure 1, the cell viability was significantly decreased in proportion to the increasing RBM and OSA concentration.

DPs cultured in 5 μg/ml OSA showed the viability reduced to 80%, addition of 50 μl/ml RBM decreased viability about 10%. In the other research revealed that the viability of RBM and OSA is more sensitive using epithelial cell than DP (Data not shown). Therefore, in this study the concentration of addition was decided at 2.4 μg/ml OSA and 30 μl/ml.

### Evaluation of RBM toxicity

In order to examine the toxicity of RBM and OSA on the DP, the DPs were cultured with As-2p (positive control), OSA (2.4 μg/ml) and RBM (30 μl/ml) without FBS for 72 h. One group was only added with As-2p, OSA, and RBM but the other group was aged by UV irradiation (312 nm) for 20 mJ/cm^2^ to further compare the negative control and experimental groups (OSA and RBM) before the injection.

**Figure 2.**
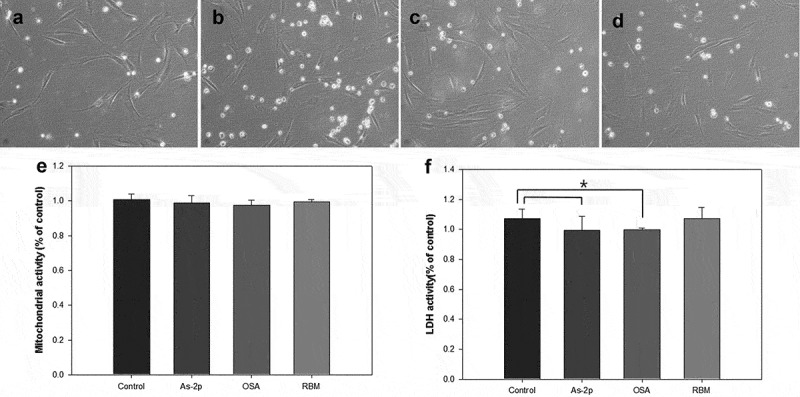
Comparison of morphology of human dermal papilla (A-D), analysis of MTT (E) and lactate dehydrogenase (LDH) activity (F). All groups were cultured in high-glucose DMEM without fetal bovine serum after exposed of UV (312 nm, 20 mJ/cm2), and then administered the various substances and evaluated at 72 h. (A) Negative control; (B) L-Ascorbic acid 2-phosphate 50 μM; (C) OSA (2.4 μg/ml); (D) Rice bran mineral extract (RBM) (30 μl/ml) (*p > 0.05).

**Figure 3.**
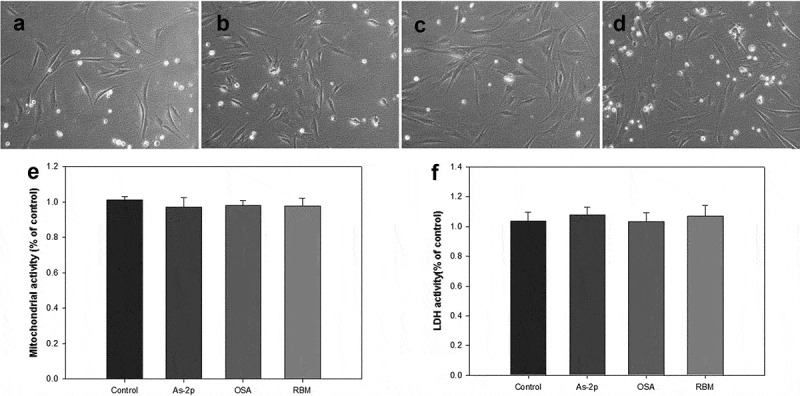
Comparison of morphology of human dermal papilla (A-D), analysis of MTT (E) and lactate dehydrogenase (LDH) activity (F). All groups were cultured in high-glucose DMEM without FBS and expose of UV, and then administered the various substances and evaluated at 72 h. (A) Negative control; (B) L-Ascorbic acid 2-phosphate 50 μM; (C) OSA (2.4 μg/ml); (D) Rice bran mineral extract (RBM) (30 μl/ml).

Morphologically, the DPs were not aged by UVA compared to Figure 2 and Figure 3 and no apoptosis and necrosis were observed. As a results of MTT assay, the viability was very similar to most of the experimental groups (control, As-2p, OSA and RBM).

One method of assaying the loss of cell membrane integrity is to measure the release of cytosolic LDH into the surrounding medium [13]. In the LDH assay, the RBM and OSA did not increased LDH activity with (Figure 2) and without UV irradiation (Figure 3). And in OSA addition some reduced LDH activity compared to control at UV irradiation but this numerical value was not considered to have a significant impact.

### Apoptosis assay of RBM

**Figure 4.**
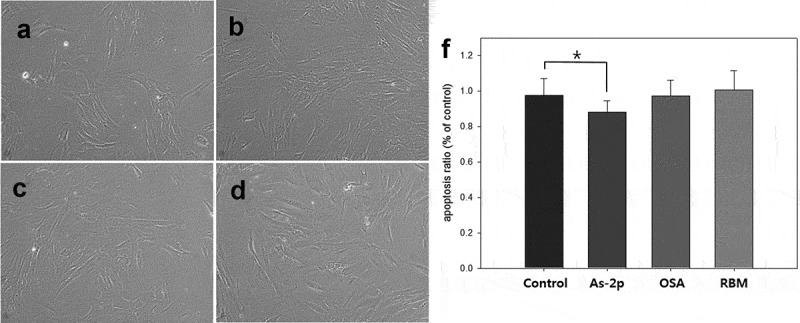
Comparison of morphology (A-D) of human dermal papilla and apoptosis assay (E). All groups were cultured in high-glucose DMEM with 10% FBS and expose of UV (312 nm, 20 mJ/cm^2^), and then administered the various substances and evaluated at 72 h. (A) Negative control; (B) L-Ascorbic acid 2-phosphate 50 μM; (C) OSA (2.4 μg/ml); (D) Rice bran mineral extract (RBM) (30 μl/ml) (*p > 0.05).

For cytotoxicity analysis, the apoptosis assay was employed. Dye is penetrated through the cell membrane of a apoptosis cell. Therefore, less dye release means less apoptosis. As shown in Figure 4, it has shown that RBM did not change of morphology and the apoptosis levels in all the experimental groups were similar to that of As-2p group. This result indicates that RBM did not induced apoptosis.

### Western blot

Dermal papilla is composed of mesenchymal cells and loose connective tissue containing type I and IV collagen and fibronectin [14] and the physicochemical nature of ECM fibrils could influence the migration and maintenance of the hair follicle [15].

**Figure 5.**
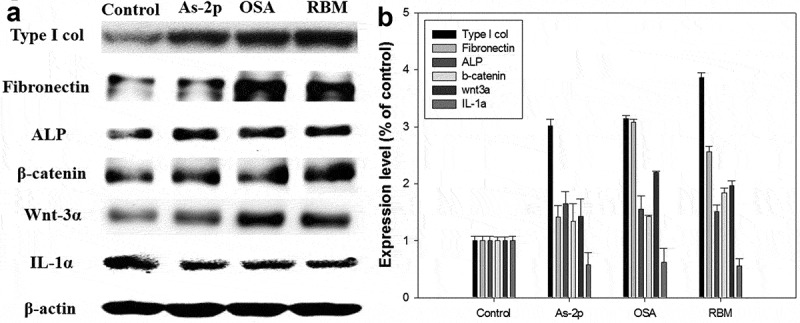
Activity of cultured dermal papilla cells injected with ortho-silicic acid (OSA) (2.4 μg/ml) and rice bran mineral (RBM) extract (30 μl/ml). (A) The protein level of cell matrix related proteins (type I collagen, fibronectin, ALP, β-catenin, and Wnt −3α) and IL-1α were examined using western blotting; (B) Graph of protein expression (with negative control level as the standard).

As shown in Figure 5, compared to the control, the cell matrix related proteins were increased by RBM and OSA. In particular, the expression of type I collagen protein was increased 400% in the RBM group. Type I collagen is one of the ECM proteins associated with the cell mechanics and collagen is the predominant component (75% of the matrix) in the ECM of the dermis [15]. However, no previous research on collagen I by the culturing of DPCs has been published. The current work showed that type I collagen expression was increased by the RBM extract. Thus, type I collagen analysis can potentially be a useful method for hair research using cultured DPCs.

In this work, when the RBM was added to the DPCs, the expression of fibronectin was increased ~2.5 times (Figure 5). Fibronectin is prevalent in the DP, both at the papilla-epidermal junction and in the DS/glassy membrane, during the anagen stage but disappears from the DP in the catagen stage and it is a well-characterized adhesive glycoprotein associated with several biological processes including cell attachment and migration [16].

Multiple abnormalities are shown during hair loss in the hair growth cycle, including curtailed anagen and premature catagen phases, and vellus hair follicles show conversion into terminal hair follicles [17]. Couchman et al reported that fibronectin was present in the DP matrix in anagen stage hair follicles and decreased in the resting stage [18]. Thus, the present results suggest that RBM and OSA could be maintained anagen phase.

And it is well known that that alkaline phosphatase (ALP) is a marker of DP cells in vivo and that enhanced ALP activity in cultured DP cells is correlated with hair regeneration. Activity of DP was moderate in very early anagen, reached a maximal level in early anagen, decreased at the proximal region of DP after mid anagen, and was kept at a low level during catagen [19]. Also, it has been reported that the early passage DP cells with high ALP activity can induce hair follicle formation when they are grafted into an ear skin wound of recipients, but, late passage DP lose ALP activity and fail to generate hair follicles [20]. Therefore, various hair inducible or inhibition materials have been evaluated by ALP analysis such as plant extract, growth factor and hormones [21–23]. In this study, OSA and RBM treatment increased ALP activity in DP cells, so this data suggest that it can be maintained anagen phase and promotes hair growth by regulating the activity of DP cells.

And it was well known that the Wnt/β-catenin-mediated signaling pathway plays a pivotal role in the regulation of hair follicle morphogenesis, hair shaft differentiation and follicular recycling [24–27]. The Wnt ligand binds to the Frizzled receptor and then activates dishevelled, which induces phosphorylation of GSK3b. As a result, b-catenin phosphorylation by GSK3b is inhibited and β-catenin is stabilized. Accumulation of β-catenin in the cytosol leads to translocation into the nucleus and activate target genes [27]. While the deletion of Wnt/β-catenin reduced the proliferation of hair follicle progenitor cells and induced the early onset of the catagen phase [28], upregulation of Wnt/β-catenin signaling resulted in a more extensive hair growth [29], especially, Wnt-3α is sufficient to maintain DP cells in the anagen state [24]. In this work, the expression of Wnt-3α and β-catenin protein was increased in OSA and RBM group compared to control group. The obtained results indicate that minerals, as a agent of rice bran extracts, might be an effective treatment for hair loss through the activation of the Wnt/β-catenin signaling pathway and subsequent cell proliferation and transcription of hair growth-related cytokines in cultured human DPCs.

Whereas, in the RBM-treated DPCs, IL-lα expression was reduced (~50%) compared to the negative control. IL-1α has been characterized as a negative hair growth regulator. In an *ex vivo* culture system, IL-1α reduced hair follicle survival, although this effect required a latency period of 2–4 days [30]. Also, IL-1α is inducible by the DP and were found in plucked hair samples of subjects with androgenetic alopecia, suggesting their participation in the pathology of male pattern baldness [31]. Based on the results, RBM inhibits IL-1α synthesis in DPCs and is expected to be useful in future hair loss prevention studies.

### RT-QPCR

**Figure 6.**
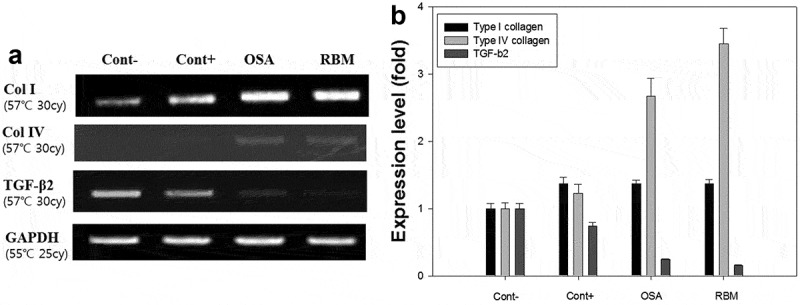
DNA expression level by western blotting in cultured dermal papilla cells injected with ortho-silicic acid (OSA) (2.4 μg/ml) and rice bran mineral (RBM) extract (30 μl/ml). (A) The mRNA level of type I collagen, type IV collagen and TGF-β2 gene; (B) mRNA expression of type I collagen, type IV collagen, and TGF-β2 (with negative control level as the standard).

The mRNA expression of the col-I gene in DPCs by RT-PCR (Figure 6) was 1.2 times higher than that of the negative control group. Expression of type IV collagen mRNA expression was also increased in the OSA- and RBM-treated DPCs, in contrast to the negative control (Figure 6). Particularly, in the RBM group, mRNA was increased ~3.5-fold relative to the positive control.

Type IV collagen is an essential component of the basement membranes of developing integumental appendages, such as teeth, feathers, and hairs [16]. Research has shown that in anagen vibrissae, as in pelage follicles, type IV collagen was evident around the internal and external follicular basement membrane zones [16] and also expressed in the outer root sheath basement membrane and in the ECM of the DP of anagen and catagen follicles [32]. In telogen follicles, where the volume of the DP ECM is much reduced, staining of the DPCs for type IV collagen was still apparent [32]. John et al reported when DPCs were initially isolated from adult rat vibrissae and cultured *in vitro*, they retained the potential to synthesize type IV collagen, but this was gradually lost in the cultures maintained for 20 days [33]. Analysis of mRNA expression in the DPCs revealed that the expression of type IV collagen was increased in the RBM group compared to the negative control group, suggesting that RBM affects the type IV collagen gene expression in DPCs.

Figure 6 shows that the mRNA of TGF-β2 was also present in the DP but at ~80% less in the RBM- and OSA-treated groups compared to the negative control. It is well known that TGF-β2 inhibits hair growth not only by reducing the anagen stage but also by promoting early entry into the catagen stage [17]. Shin et al showed that ginsenoside F2 increased the proliferation of HHDPCs and reduced expression of TGF-β2 related factors and sterol regulatory element binding protein (SREBP) cleavage-activating proteins (SCAPs) involved in dihydrotestosterone-induced hair loss *in vitro* [33]. Furthermore, expression of TGF-β2 was 51 and 72% lower than the control in the groups treated with 0.1 and 1 µM ginsenoside, respectively [17]. Kang et al reported that the ethanol extract of *Schisandra nigra* increased the hair fiber length of rat vibrissa follicles. In that work, the vibrissa follicles in the anagen phase were treated with the *S. nigra* extract for 7 days and TGF-β2 expression in the bulb area was found to be lower than that of the control follicles which were expected to be in the anagen-catagen transition phase [34]. The authors concluded the extract had the potential to promote hair growth via downregulation of TGF-β2 and the proliferation of DP [34]. In our experiment, the RBM extract inhibited TGF-β2 gene activity in the DPCs, suggesting that the RBM extract also has the possibility to promote hair growth.

### Immunohistochemistry

**Figure 7.**
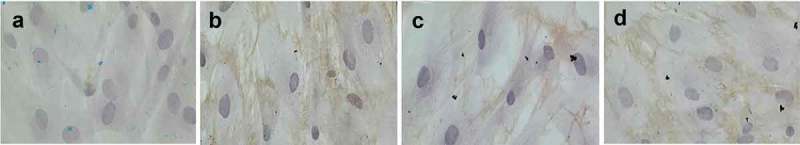
Immunohistochemistry of versican in cultured dermal papilla cells after injection with treatment substance at 72 hour. (A) Negative control; (B) Positive control; (C) Ortho-silicic acid (OSA) (2.4 μg/ml); (D) Rice bran mineral (RBM) extract (30 μl/ml).

Immunohistochemical staining was performed to detect versican expression (Figure 7). Compared to the negative control, versican was expressed more in the OSA-treated DPCs and, particularly, in the RBM-treated DPCs. Versican is a hair follicle formation related proteoglycan [35] found in the ECM [36,37]. According to Diana et al, versican is a widespread ECM component of rodent and human skin, with a unique pattern of distribution in murine hair follicle morphogenesis and cycling. Additionally, veriscan is most abundant in the dermal matrix, particularly in the human reticular dermis, and in the matrix of follicular DP [38]. *Ecklonia cava*, a seaweed found in the sea around Korea and Japan, has anticoagulant, antioxidant, anticancer and matrix metalloproteinase (MMP) inhibitory activities [39–41]. When the *E. cava* extract fraction was added to DPCs, the proliferation was increased [41] and the expression of the versican mRNA was increased ~1.2-fold [42]. Diana et al found that versican immunostaining decreased by retreating down the papillae until in the telogen (resting) phase, all staining for versican had disappeared from the dermal cells [38]. Therefore, the RBM seems to have influenced the delay of the DPCs entering the catagen stage.

## Conclusion

RBM may prolong the anagen phase through increasing of type I collagen, fibronectin, ALP, type IV collagen and versican through by activation of the β-catenin/Wnt signaling pathway. In addition, RBM was shown to reduce the expression of inflammation-related markers (IL-1α and TGF-β2) in cultured DPC. Therefore, it is considered that RBM may be particularly useful as a natural ingredient for hair loss prevention and hair growth enhancement.
